# Analyzing the Surface Topography of Hafnium Nitride Coating on Titanium Screws: An In Vitro Analysis

**DOI:** 10.7759/cureus.57385

**Published:** 2024-04-01

**Authors:** Shilpa M Jose, Vaishnavi Rajaraman, Padma Ariga, Dhanraj Ganapathy, Saravanan Sekaran

**Affiliations:** 1 Prosthodontics, Saveetha Dental College and Hospitals, Saveetha Institute of Medical and Technical Sciences, Saveetha University, Chennai, IND

**Keywords:** uncoated, titanium implants, surface topography, field emission-scanning electron microscope, hafnium nitride, elements, energy-dispersive x-ray spectrometry, coated dental implant

## Abstract

Background

The use of surface coatings to enhance the properties lacking in titanium has attracted significant focus in recent times. Hafnium nitride (HfN) coatings could be explored as promising in the osteoinductive properties of titanium implants. HfN exhibits excellent mechanical attributes, such as hardness and wear resistance, and is often used as a coating on high-end equipment for protection. The findings from this research may carve a new path for the production and optimization of HfN coatings to enhance the longevity and augment properties of implant materials. Thus, the present study was orchestrated to elucidate the surface morphology of HfN coating, ultimately contributing to the advancement of dental implant biomaterials.

Materials and methods

A total of twenty samples of medical grade commercially pure titanium screws (2 mm diameter and 7 mm length) were procured from G. R. Bioure Surgical System Pvt. Ltd., Ravali, Uttar Pradesh, India, and ten samples were reacted with HfN (0.1 M) (Nano Research Elements, Kurukshetra, Haryana, India) in 100% ethanol and stirred continuously for about 48 hours. Then these screw samples were immersed in the prepared colloidal suspension and sintered for two hours at 400 degrees centigrade. The implant screws were affixed onto metal supports. The magnifications for photomicrographs at ×30, ×200, ×1,500, ×3,000, and ×5,000 were standardized. Elementary semi-quantitative analysis of both dental implants was conducted using energy-dispersive X-ray spectrometry (EDX) coupled with the field emission scanning electron microscope (FE-SEM) equipment (JEOL Ltd., Akishima, Tokyo, Japan). The software used for the analysis of the obtained images is SEM Center.

Results

The surface analysis using the scanning electron microscope (SEM) showed the coating of HfN over titanium screws. The difference in surface morphology of both the group of implant screws can be visualized under 40.0 and 10.0 mm working distance (WD) for both groups. The surface analysis using the EDX of uncoated titanium screws shows five elements in the spectrum: titanium (Ti), oxygen (O), aluminum (Al), carbon (C), and vanadium (V). The EDX of the HfN-coated screws has two additional metals dispersed in the spectrum, hafnium (Hf). The element characteristics are tabulated with their apparent concentration, k ratio, line type, weight percentage, standard label, and factory label for uncoated titanium screws and HfN-coated titanium screws.

Conclusion

The study evaluated HfN coating over medical grade commercially pure titanium. The surface topography of coated versus uncoated was visualized. The scanning electron microscope (SEM) images showed a homogenous coating over the titanium surfaces, and the EDX showed elemental dispersion of the coated implant. The study aims to provide a comprehensive understanding of the coating's surface morphology, which will aid in the development of more durable and biocompatible implants. This thereby provides a promising scope for further research of this novel metal coating for use in the biomedical sectors, specifically for dental implants.

## Introduction

Over decades, titanium has been one of the most commonly used prosthetic biomaterials [1]. Often considered the gold standard, titanium has its own disadvantages in terms of cost, stress shielding, radiographic imaging, potential for corrosion, etc. [2]. These challenges led to the idea of surface coatings over titanium to improve the mechanical, biological, and physical properties of titanium alloy [3]. Surface coatings have been widely researched in recent times to alleviate problems associated with titanium implants [4,5].

The use of surface coatings to enhance the properties lacking in titanium has attracted significant focus in recent times. The properties it aims to improve are corrosion resistance, biocompatibility enhancement, osseointegration promotion, reduced wear and friction, anti-bacterial properties, and improved imaging [6,7]. Overall, surface coatings over titanium implants offer a versatile approach to enhance their performance and address specific challenges associated with implantology, ultimately improving patient outcomes.

The periodic table element, hafnium, falls into the same period, similar to standard titanium. Belonging to period 6 on the periodic table, hafnium has been researched as an alloy combination or coating in various in vitro scenarios [8-10]. Hafnium is also similar to titanium in its behavior with osseous tissues [8,9]. This hafnium metal coating of commercially available titanium implants has proven to show some superior properties to titanium with in vitro studies and soft tissue [11-13]. Hafnium nitride (HfN) coatings could be explored as promising in the osteoinductive properties of titanium implants [4, 14-16].

HfN exhibits mechanical attributes such as hardness and wear resistance and is used as a coating on high-end equipment for protection [17,18]. However, there is a lack of literature on this novel coating HfN, requiring basic to advanced evaluations of their surface topography and other properties favorable for dental implantology [15]. The findings from this research may carve a new path for the production and optimization of HfN coatings to enhance the longevity and augment properties of implant materials.

This research carries significant weight for the development of a new coating, HfN, over titanium screws. By comprehensively evaluating the surface topography of HfN-coated titanium screws, this study aims to advance our understanding of protective coating technologies for biomedical applications. Our investigation aims to elucidate the surface morphology of HfN coating, ultimately contributing to the advancement of dental implant biomaterials.

## Materials and methods

**Figure 1:**
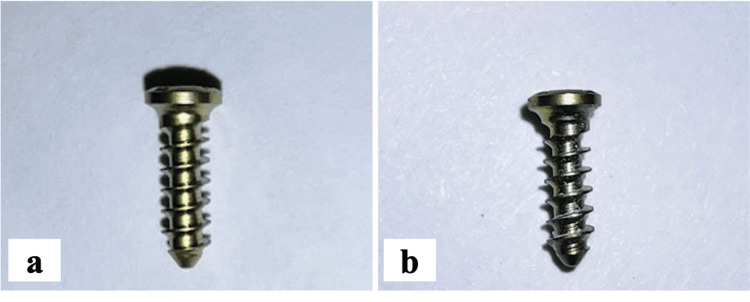
The coated and uncoated titanium screw samples a: Hafnium nitride (HfN)-coated titanium screws; b: Medical grade commercially pure titanium screws Image credit: Rajaraman et al. [15]

The design of the current original research was executed in the White Lab, Research Cell, in the university set-up of Saveetha Dental College, India, after approval from the Institutional Review Board for research, with allocated project number SRB/SDC/UG-1701/23/PROSTHO/016. A total of twenty samples of commercially pure titanium screws (2 mm diameter and 7 mm length) were procured from G. R. Biocure Surgical System Pvt. Ltd., Ravali, Uttar Pradesh, India. Ten samples were reacted with HfN powder (Nano Research Elements, Kurukshetra, Haryana, India), 0.1 M in 100% ethanol, and stirred continuously for about 48 hours. Then these screw samples were immersed in the prepared colloidal solution and sintered for two hours at 400 degrees centigrade [19]. The coated screw thus obtained was used as the test group, and uncoated titanium screws were used as the control group (Figure 1a and Figure 1b).

**Figure 2:**
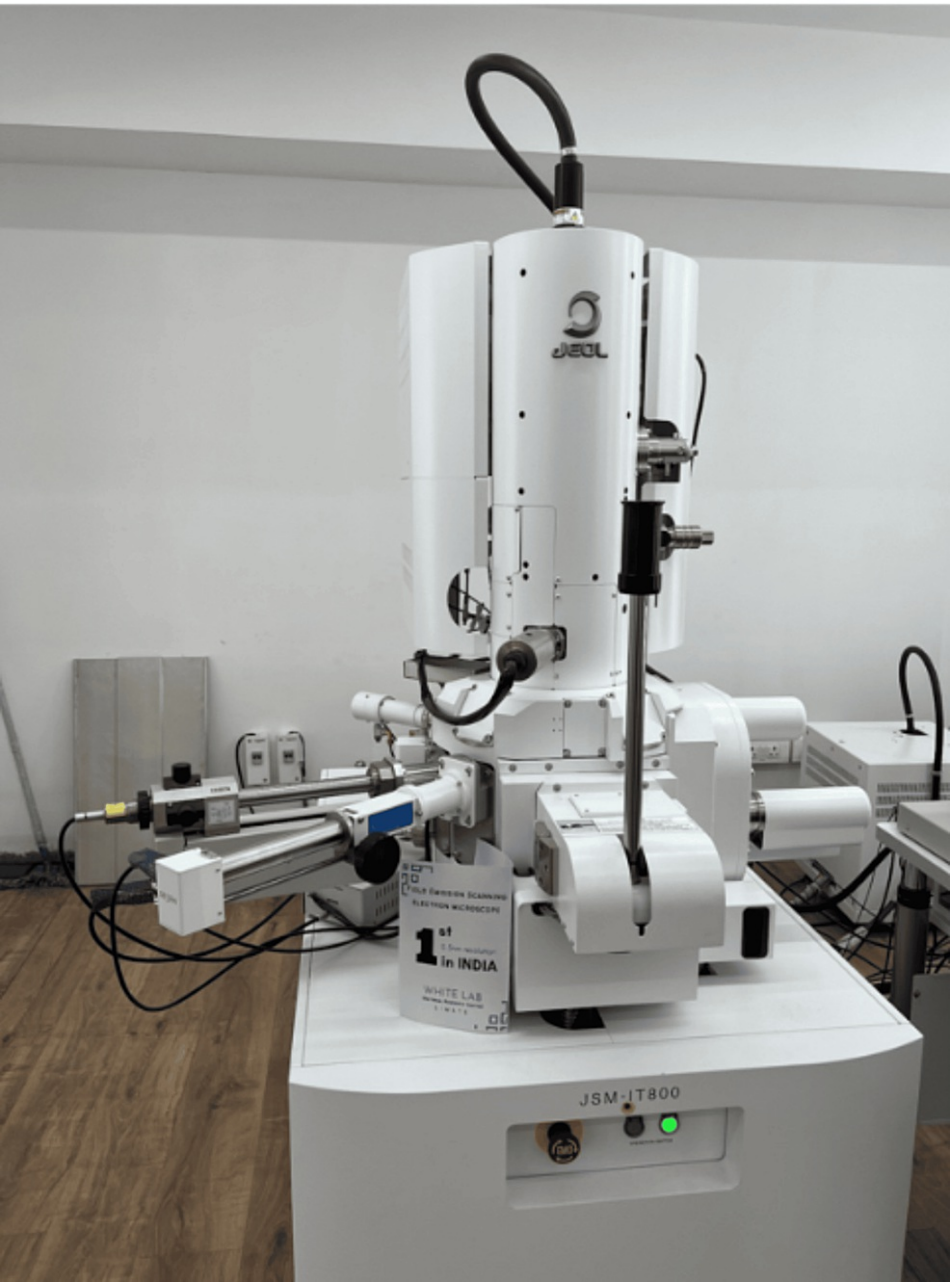
A field emission scanning electron microscope with a comprehensive suite of analytical capabilities, including the Oxford Instruments Ultim Extreme detector, JEOL IT800 FE-SEM®

The titanium implant screws, both coated and uncoated, were affixed onto metal supports using double-sided carbon tape. The magnifications for photomicrographs at ×30, ×200, ×1,500, ×3,000, and ×5,000 were standardized, except for specific regions of interest. Surface characteristics were visualized using the JSM IT800 FE-SEM® by JEOL Ltd., Akishima, Tokyo, Japan (Figure 2), an FE-SEM contained with the Oxford Instruments Ultim Extreme Detector. The SEM was taken in 100- and 5-μm sections for both uncoated and coated groups, each at 40 and 10 mm working distance.

This state-of-the-art ultrahigh-resolution FE-SEM boasts advanced high-resolution analytical technology. The microscope has a resolution of 0.7 nm (20 kV), magnification of ×10-×2,000,000, and acceleration voltage of 0.01-30 kV. The software used for the analysis of the obtained images is SEM Center.

Elementary semi-quantitative analysis of both dental implants was conducted using energy-dispersive X-ray spectrometry (EDX) coupled with the FE-SEM equipment. The implants were secured onto the same holder as before, and various regions were randomly analyzed using the EDX system, which automatically detected surface elements. The resulting data represent the weight percentage (wt%) of each element. The research utilized the sophisticated model X-Plor-30/C-Swift with Aztec software for viewing surface topography. 

## Results

**Figure 3:**
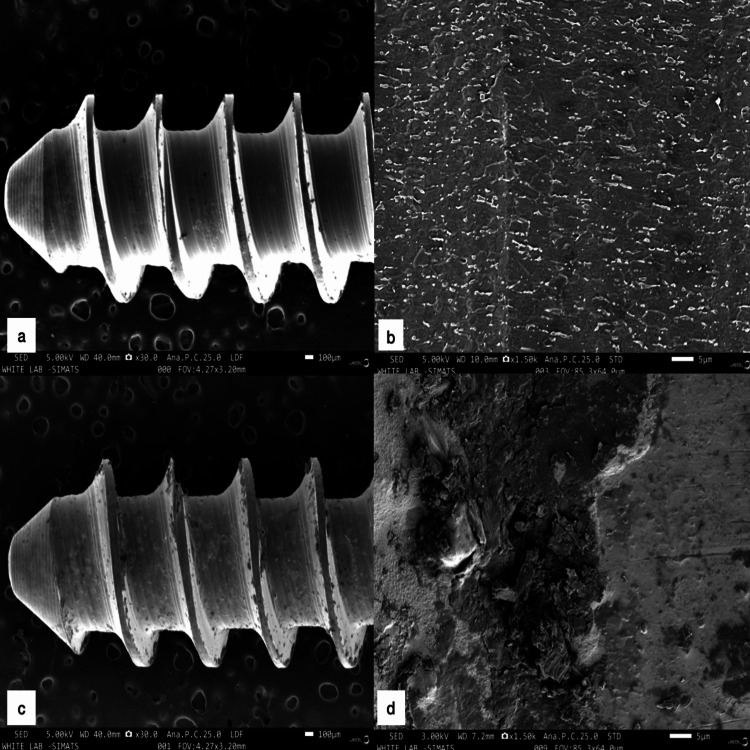
The surface analysis images of the uncoated and coated titanium screws using a scanning electron microscope a: Uncoated titanium screws in 100 µm sections under 40.0 mm working distance (WD); b: Uncoated titanium screws in 5 µm sections under 10.0 mm WD; c: Hafnium nitride-coated titanium screws in 100 µm sections under 40.0 mm WD; d: Hafnium nitride-coated titanium screws in 5 µm sections under 10.0 mm WD

The surface analysis using the SEM showed the coating of HfN over titanium screws. The difference in surface morphology of both the group of implant screws can be visualized in various sections (Figure 3).

**Figure 4:**
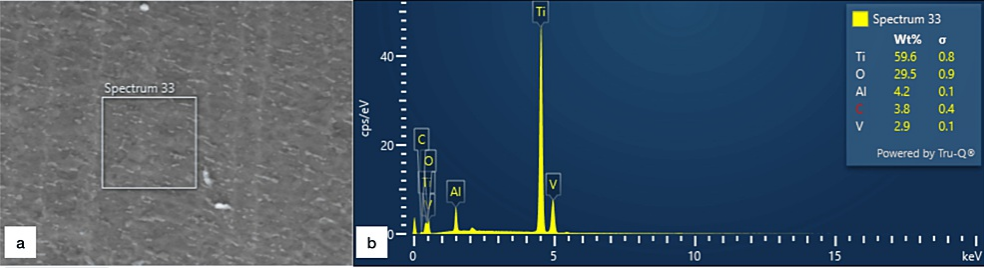
Energy-dispersive X-ray spectrometry of uncoated titanium screws a: Electron image in spectrum 33; b: Graph showing elemental distribution in the spectrum: titanium (Ti), oxygen (O), aluminum (Al), carbon (C), and vanadium (V) cps/eV, counts per second per electron-volt

The surface analysis using the EDX of uncoated titanium screws shows five elements in the spectrum: titanium (Ti), oxygen (O), aluminum (Al), carbon (C), and vanadium (V) (Figure 4).

**Figure 5:**
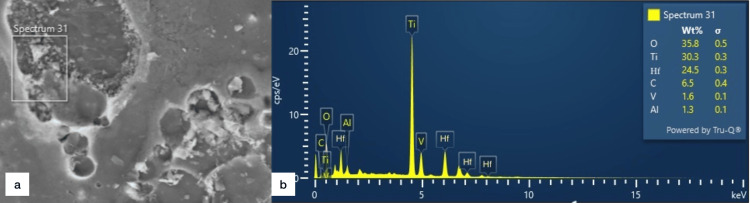
Energy-dispersive X-ray spectrometry of hafnium nitride-coated titanium screws a: Electron image in spectrum 31; b: The graph showing elemental distribution in the spectrum: titanium (Ti), oxygen (O), aluminum (Al), carbon (C), and vanadium (V) cps/eV, counts per second per electron-volt; hf, hafnium

The EDX of the HfN-coated screws has two additional metals dispersed in the spectrum, hafnium (Hf) (Figure 5). 

The element characteristics are tabulated with their line type, apparent concentration, k ratio, weight percentage, standard label, and factory label for uncoated titanium screws (Table 1).

**Table 1:** The element characteristics of uncoated titanium screws with their line type, apparent concentration, k ratio, weight percentage, standard label, and factory label

Element	Line Type	Apparent Concentration	k Ratio	wt%	wt% Sigma	Standard Label	Factory Standard
Ti	K Series	15.47	0.15471	59.60	0.77	Ti	Yes
O		1.31	0.00441	29.41	0.86	SiO_2_	
Al		0.77	0.00553	4.18	0.09	Al_2_O_3_	
C		0.16	0.00158	3.84	0.45	C	
V		0.74	0.00738	2.87	0.12	V	

The element characteristics were tabulated with their line type, apparent concentration, k ratio, weight percentage, standard label, and factory label for HfN-coated titanium screws (Table 2). 

**Table 2:** The element characteristics of hafnium nitride-coated titanium screws with their line type, apparent concentration, k ratio, weight percentage, standard label, and factory label

Element	Line Type	Apparent Concentration	k Ratio	wt%	wt% sigma	Standard Label	Factory Standard
Ti	K Series	7.34	0.07336	30.26	0.28	Ti	Yes
O		2.70	0.00910	35.84	0.47	SiO_2_	
Al		0.19	0.00138	1.31	0.06	Al_2_O_3_	
C		0.26	0.00264	6.49	0.45	C	
V		0.40	0.00401	1.64	0.09	V	
Hf		4.99	0.04987	24.46	0.29	Hf	

## Discussion

In this research, we have studied the surface coating of a novel metal called HfN over titanium screws. The surface coating of HfN over titanium screws was homogenous. This was substantiated by the SEM sections obtained as images in 40 and 10 mm working distance. The elemental distribution of the coated versus uncoated screws was tabulated in detail. Additionally, the study considers the material characteristics and composition of the innovative coating. Elemental analysis, surface morphology assessments, and microstructural examinations are conducted. 

Studies state that surface topography affects bone response at the micrometer or nanometer level [20,21]. Implant surface topography is not well researched or consistently reported in experimental research. Another drawback is that many in vitro evaluations do not predict or correlate with in vivo performance. This said, in a few culture models, surface characterization positively affects osteogenic cellular activities [4,22,23]. 

Researchers have found that scanning electron microscopy and energy dispersive X-ray spectroscopy analysis on physical vapor deposition of zirconium nitride in titanium screws were effective, and preosteoblasts were seeded onto the covering and showed better adhesion of particles than the uncoated titanium screws [24]. Surface modification of titanium implants that offer both osteoinductive and antimicrobial properties can be generated. 

Titanium implant surfaces, namely, titanium dioxide nanotubes and titanium oxide grit blasted surfaces, were investigated for bone bonding and showed that the titanium-coated nanotubes improve osteoblast proliferation and adhesion [25,26]. Titanium implants coated with graphene oxide were evaluated for osseointegration by culturing bone marrow mesenchymal stem cells (BMSCs), and the results showed that the BMSCs cultured on the titanium screws coated with graphene oxide exhibited the best adhesion, proliferation, and differentiation, compared with that of uncoated titanium screws [27,28]. A study conducted by Svensson et al. evaluated osseointegration with an antimicrobial nanostructured noble metal coating over titanium, and the results demonstrate that it promotes osseointegration [28,29]. Therefore, it could be used to add extra implant functionality to increase resistance to infection without the use of antibiotics.

The surface morphology of implant coatings plays a crucial role in dental implantology due to their clinical relevance in enhancing osseointegration and long-term implant success. By influencing factors such as surface roughness, topography, corrosion, and wettability, HfN coatings over titanium can promote favorable interactions with surrounding tissues, leading to improved implant stability and reduced risk of peri-implant complications [15]. Hence, this study concentrated on analyzing the surface topography of the HfN-coated titanium implants. However, translating laboratory findings to clinical practice involves several considerations. Regulatory approval, such as from the FDA or equivalent agencies, is necessary to ensure the safety and efficacy of novel surface coatings. Challenges may arise in maintaining consistency between laboratory conditions and real-world healthcare settings, as well as in standardizing manufacturing processes to meet regulatory requirements. Despite these challenges, the potential benefits of HfN coatings in enhancing dental implant outcomes underscore the importance of ongoing research and collaboration between researchers, clinicians, and regulatory bodies to facilitate their implementation in clinical practice.

Limitations

The interplay between the coating and the titanium substrate is crucial, as any potential interface weaknesses could compromise the overall corrosion resistance of the implant. Furthermore, the biological aspect of corrosion resistance has not been explored. The study does not delve into the biocompatibility of the coating, evaluating its impact on cell viability, adhesion, and proliferation. This is essential to ensure that the corrosion-resistant coating not only performs well in simulated environmental conditions but also maintains a favorable interaction with the surrounding biological tissues.

## Conclusions

In conclusion, this study evaluated HfN coating over medical grade commercially pure titanium. The surface topography of coated versus uncoated was visualized. The SEM images showed a homogenous coating over the titanium surfaces, and the EDX showed elemental dispersion of the coated implant. The study provided a comprehensive understanding of the coating's surface morphology, aiding in the development of more durable and biocompatible implants. This thereby provides a promising scope for further research of this novel metal coating for use in the biomedical sectors, specifically for dental implants.
